# Mayaro Virus: An Emerging Alphavirus in the Americas

**DOI:** 10.3390/v16081297

**Published:** 2024-08-14

**Authors:** Lily Li Lin Wei, Rufaro Tom, Young Chan Kim

**Affiliations:** 1 Somerville College, University of Oxford, Woodstock Road, Oxford OX2 6HD, UK; lily.wei@some.ox.ac.uk (L.L.L.W.); rufaro.tom@some.ox.ac.uk (R.T.); 2 Oxford Vaccine Group, Department of Paediatrics, University of Oxford, Oxford OX3 7LE, UK; 3 Centre for Human Genetics, Division of Structural Biology, University of Oxford, Roosevelt Drive, Oxford OX3 7BN, UK

**Keywords:** Mayaro virus, alphavirus, arbovirus, arthralgia, public health, vaccines

## Abstract

Mayaro virus (MAYV) is an arbovirus first isolated in Trinidad and Tobago in 1954. MAYV is the causative agent of Mayaro fever, which is characterised by high fever, maculopapular rash, myalgia and arthralgia. The potential for chronic arthralgia is of particular clinical concern. Currently, MAYV outbreaks are restricted to South and Central America, with some cases reported in Africa as well as several imported cases in Europe. However, in recent years, MAYV has become a growing global concern due to its potential to emerge into urban transmission cycles. Challenges faced with diagnostics, as well as a lack of specific antivirals or licensed vaccines further exacerbate the potential global health threat posed by MAYV. In this review, we discuss this emerging arboviral threat with a particular focus on the current treatment and vaccine development efforts. Overall, MAYV remains a neglected arbovirus due to its limited area of transmission. However, with the potential of its urbanisation and expanding circulation, the threat MAYV poses to global health cannot be overlooked. Further research into the improvement of current diagnostics, as well as the development of efficacious antivirals and vaccines will be crucial to help prevent and manage potential MAYV outbreaks.

## 1. Introduction

First isolated in Trinidad and Tobago in 1954, Mayaro virus (MAYV) is a single-stranded positive-sense RNA arbovirus belonging to the *Alphavirus* genus and *Togaviridae* family [1]. MAYV is transmitted by *Haemagogus janthinomys* mosquitoes [2], with outbreaks largely restricted to South and Central America [3,4,5]. However, as MAYV has been isolated from other genera of mosquitoes, including *Aedes Aegypti*, there is growing concern that MAYV could adapt and emerge into urban transmission cycles [6,7,8]. Moreover, MAYV is the causative agent of Mayaro fever, an acute febrile illness characterised by high fever, arthralgia and maculopapular rash [9]. In some cases, this can progress into chronic debilitating arthralgia and neurological complications [9,10]. Subsequently, there is a pressing need to develop novel diagnostic methods and therapeutics to prevent MAYV spread. Currently MAYV is recognised as a neglected tropical disease, and there is a lack of available therapeutics against MAYV. This review details the current literature on MAYV; in particular, the current progress on MAYV therapeutics and vaccine development will be explored.

## 2. MAYV Structure and Genome

**Figure 1:**
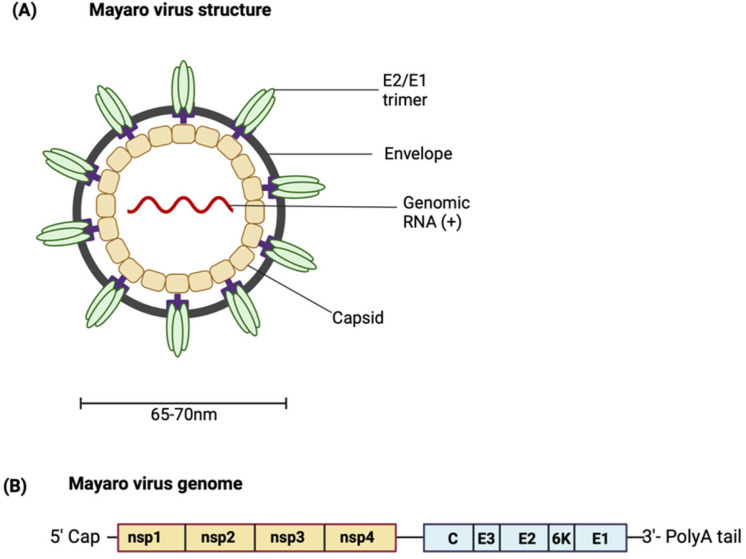
(**A**) Structure of MAYV. (**B**) Schematic representation of the MAYV genome. (Created with BioRender.com.

MAYV is a positive-sense single-stranded RNA icosahedral enveloped virus (Figure 1A). The 11.5 kb genome encodes two open reading frames (ORFs) [11,12]. The ORFs are separated by an intergenic region containing an internal ribosome entry site (IRES). The IRES aids in the generation of subgenomic RNA using internal elements or a subgenomic promoter). The IRES is also the start site and leader sequence for the 26S mRNA (Figure 1B). The first ORF encompasses the 5′ two-thirds of the genomic DNA and encodes a non-structural polyprotein [13]. The non-structural polyprotein is proteolytically cleaved into non-structural proteins (nsPs) 1–4 [14]. As limited studies characterising MAYV proteins have been conducted, most knowledge regarding the role of MAYV proteins is based on findings from closely related alphaviruses. Each nsP has a distinct function; the role of each nsP in alphavirus replication and pathogenesis is summarised in Table 1 [15,16,17,18,19,20,21,22,23]. For more details about the functions of the nsPs in the alphavirus life cycle, see the comprehensive reviewed by Mota et al. [13].

The second ORF encodes the structural polyprotein, which is processed to produce the structural proteins—the capsid (C) protein, the envelope glycoproteins E1, E2 and E3, as well as the small 6K/transframe (TF) protein. The viral particle is made up of copies of the capsid protein associated with the RNA genome. This forms an icosahedral nucleocapsid which is enclosed in the viral envelope derived from the host membrane and studded with viral E1 and E2 transmembrane glycoproteins arranged in heterodimeric trimers [24]. The envelope glycoproteins are an attractive target for designing vaccines capable of eliciting neutralising antibodies [25]. E1 is a membrane fusion protein. E2 and E3 are produced from the cleavage of precursor E2 (pE2) by a furin-like protease. E2 is involved in receptor binding, and while the function of E3 has not been well characterised, it is hypothesised to contain signal sequences for the insertion of polypeptides into the endoplasmic reticulum for further processing [13,26]. The 6K protein has been shown to have properties typical of a viroporin, increasing membrane permeability and creating conditions that favour virus budding [27]. It is suggested that an interaction between E2 and 6K proteins may be necessary for the interaction of E2 with the capsid protein, hence highlighting the role of the 6K protein in mediating viral budding [28,29]. Similar to the 6K protein, the TF protein is suggested to be involved in virus assembly and budding, although the differences in their exact functions are still being investigated [29].

## 3. A History of MAYV Transmission: Phylogenetics and Epidemiology

### 3.1. Phylogenetics

MAYV can be considered a monophyletic group; this group can be divided into two major genotypes and one minor genotype [30]. The major genotypes include genotype D, which is widely dispersed and includes the majority of isolates from 1954 to 2003 distributed in South America and Caribbean, and genotype L, which is limited to isolates found in Brazil and Haiti [31]. A new clade, genotype N, composes the minor genotype and consists of a single sequence isolated from Peru in 2010. Serological and sequencing studies have classified Mayaro virus as part of the Semliki antigenic complex, which is composed of eight viruses of veterinary and medical importance [32]. Other viruses in the Semliki complex include the Bebaru, Chikungunya, Getah, Semliki Forest, Ross River, O’nyong-nyong and Una viruses; these are all characterised by skin rash and arthritis. Subsequently, it shares common antigenic sites with other viruses in the Semliki complex, leading to the possibility of cross-reactivity with polyclonal immune sera among species [33].

### 3.2. Transmission Mechanisms

MAYV is transmitted through the bite of a female mosquito from the genus *Haemagogus* [2]. This is also a vector in yellow fever and can infect, replicate and disseminate in both vertebrates and invertebrate hosts. There is evidence that mosquitos from the genus *Aedes* could be involved in the transmission of Mayaro virus and could subsequently contribute to the establishment of Mayaro fever outbreaks in Brazil, where *Aedes aegypti* and *Aedes albopictus* are present [34]. *Aedes aegypti* is highly anthropophilic and has a nearly worldwide distribution, although this vector has not been reported in Europe. However, due to climate change and global warming, the distribution of *Ae. aegypti* could expand [35]. Furthermore, in 2017, isolates of MAYV from two pools of adult *Ae. aegypti* demonstrated the vertical transmission of MAYV [36]. This mechanism could potentially contribute to the maintenance of the arbovirus during interepidemic periods as well as the general spread of the arbovirus. In comparison, *Ae. albopictus* is native to Southeast Asia, and in recent years, it has begun to establish itself in several regions across the world [34]. Interestingly, a case of airborne transmission has been reported in a laboratory worker [37].

### 3.3. Epidemiology

Mayaro virus was first isolated in 1954 in Trinidad and Tobago [7]. Since it was first isolated, small outbreaks of Mayaro virus have been reported in South America and Central America, with most outbreaks occurring in Brazil [4]. Most cases have been restricted to regions near tropical forests. However, the urban *Aedes aegypti* have been shown to be competent vectors, highlighting the concern that MAYV could emerge into an urban transmission cycle, further increasing the demographic at risk [6,7,8]. Clinical presentations of cases have included arthralgia, fever, headache and myalgia. As MAYV has very similar presentations to other mosquito-borne viruses, including dengue virus (DENV) and Chikungunya virus (CHIKV), it is likely that MAYV cases have been underreported due to misdiagnosis. Moreover, as cases, such as in Haiti 2015, have been reported which are not associated with the forest, this indicates that MAYV could be entering urban cycles.

#### 3.3.1. Brazil

Several MAYV cases have been identified in Brazil [4]. In particular, cases have been reported in the Amazon region and Mato Grosso. MAYV has the capability of causing relatively large outbreaks. Cases of MAYV were recorded from December 1977 to June 1978 in Belterra in Para. Approximately 800 individuals had a suspected MAYV infection; this represented 20% of the population in that area. Moreover, isolates from confirmed human cases and vectors led to the characterisation of MAYV as genotypes L and D [4,30,31]. This highlights the potential for MAYV to cause large outbreaks. Several other MAYV outbreaks have occurred in Brazil. In 2008, a MAYV outbreak occurred in Pau D’arco in Para [38]. Many residents in this region resided in the middle of the forest. Azevedo et al. (2009) conducted house to house surveys [39]—105 people were found to have had febrile illness in the past 30 days or had contact with someone with febrile illness. Thirty-six people were reported to be positive for IgM against MAYV. They found that 33 individuals were symptomatic, with fever reported in all patients. Moreover, the majority of symptomatic individuals reported myalgia, arthralgia, headaches and articular oedema [39].

MAYV cases in other parts of Brazil have also been reported. During a DENV outbreak in Sinop, Mato Grosso, six cases seropositive for MAYV were identified. These cases had similar presentations to DENV infections; this further highlights the difficulty in differentiating between these arbovirus infections [40]. Subsequently, it is likely that underdiagnosis of MAYV may occur due to the misdiagnosis of MAYV in areas where another arbovirus is endemic.

#### 3.3.2. Haiti

The first evidence of MAYV in Haiti was found in 2014. In a study, 252 school children presenting with acute undifferentiated febrile illness in rural areas of Haiti between May and August 2014 were tested, and two MAYV cases were identified [41]. RT-PCR found that among the 82 children positive for CHIKV, 1 was also positive for MAYV. In the same cohort of school children, the second MAYV case identified was also infected with DENV1 [42]. This patient was an 8-year-old boy from a non-forest area, who initially presented with fever, abdominal pain and a high temperature. MAYV infection was confirmed following a blood sample being tested using RT-PCR. Further tests identified MAYV as genotype L [42]. As this child was from a non-forest area and experienced co-infection with DENV-1, it is suggested this could represent transmission of MAYV through the mosquito vector *A. aegypti* [41,42].

#### 3.3.3. Venezuela

The first MAYV cases in Venezuela were reported in Miranda State in 2000 [30]. As detected by indirect ELISA for IgM and IgG, three out of four members of a family were seropositive for MAYV. This family initially presented with fever, malaise, myalgia, headache and malaise. Two weeks following the initial onset of disease, cases presented with severe joint symptoms and lower limb hyperesthesia. The source of infection was believed to be mosquitoes as clinical presentations occurred following a dinner near the Padrón agriculture station, where the cases reported being frequently bitten by mosquitos [30].

In 2010, a larger outbreak of MAYV occurred in La Estación, Portuguesa State, in Venezuela [43]. A total of 77 cases were reported, with 19 cases confirmed seropositive. This outbreak had a larger proportion of female cases. Using genome sequencing, genotype D MAYV was reported as the cause of the outbreak [30].

#### 3.3.4. French Guiana

The first case in French Guiana was identified in 1996 using immunofluorescent antibody testing with a specific mouse antibody [44]. Between 2017–2019, 1–3 cases of MAYV were reported in French Guiana each year. In 2020, 13 lab-confirmed cases were reported [45].

#### 3.3.5. Ecuador

In 1997, the first evidence of MAYV in Ecuador was reported. A cross-sectional seroepidemiological study of 338 subjects reported that some subjects were MAYV seropositive [46]. Subjects were based in an Amazonian military base and diagnosed using ELISA tests. Amazonian natives were found to be 20 times more likely to have been exposed to MAYV compared with subjects from other regions. Moreover, this study identified two risk factors for MAYV in this population; these included age and hunting. Subjects who hunted in the forest and who were over the age of 30 had a higher probability of MAYV seropositivity. As age is thought to be indicative of the duration of residence in the rainforest, this could indicate that a MAYV outbreak may have occurred 2 decades prior when the younger people were not hunting.

#### 3.3.6. Europe

Imported cases have been reported in European countries, including in the Netherlands, France and Germany [4]. These cases have been reported in travellers returning from countries in South America. In the Netherlands in 2008, the first imported case reported was from Surinam. In 2013, an imported case from Brazil was also reported in the Netherlands [47].

## 4. Alphavirus Life Cycle

**Figure 2:**
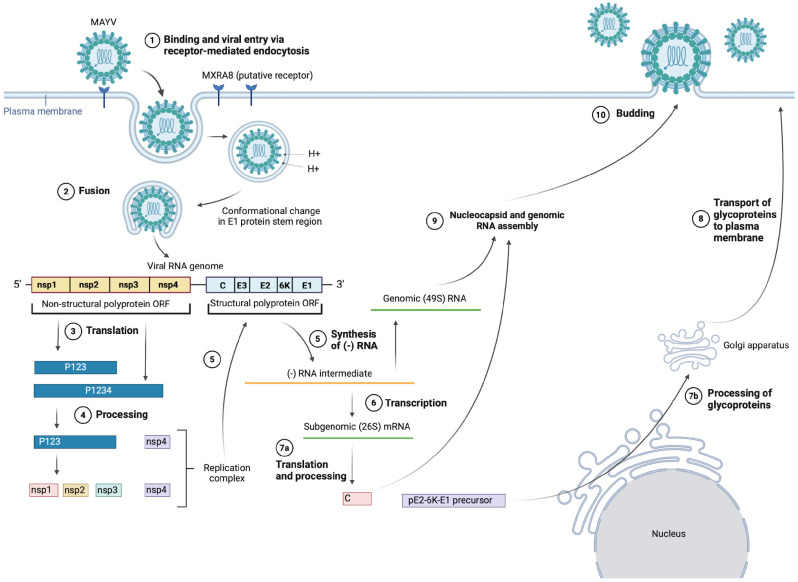
MAYV entry, replication, assembly and budding. (Created with BioRender.com).

There is limited research on the MAYV-specific life cycle; hence, the information presented in the following section is inferred from literature regarding the life-cycle stages of other alphaviruses [48,49] as well as some MAYV-specific studies, such as Ribeiro-Filho et al.’s 2021 paper presenting the cryo-EM structure of MAYV and their findings about MAYV protein interactions [24], as shown in Figure 2. In particular, we will discuss cell entry due to the important role of the E1 and E2 glycoproteins as vaccine immunogens. For further details on replication, assembly and budding, the alphavirus life cycle has been reviewed comprehensively by Skidmore and Bradfute (2023) [50].

It is suggested that MAYV enters target cells via receptor-mediated endocytosis, mainly through clathrin-coated pits, but also alternatively through caveolin-coated pits [51]. The exact details of this interaction are still unclear, but there is evidence suggesting that cell entry is mainly mediated through the viral E2 surface glycoprotein binding to complementary host cell receptors, with the putative host receptor being Mxra8, a cell adhesion protein also known as DICAM, ASP3 and limitrin [52]. Zhang et al. (2018) showed that Mxra8 deletion in 3T3 and MEF cells led to reduced infectivity of MAYV compared with the control. Further experimentation with CHIKV (closely related to MAYV) also showed that Mxra8 bound directly to CHIKV particles and enhanced viral attachment and entry. Furthermore, administration of anti-Mxra8 monoclonal antibodies decreased CHIKV titres significantly, establishing Mxra8 as a potential entry mediator for arthritogenic alphaviruses, including MAYV, CHIKV and Ross River virus (RRV) [52].

Following endocytosis, the viral particle is found within an endosome, where the action of ATP-dependent proton pumps causes acidification of the vesicle, enabling fusion of the viral and endosomal membranes [28,53]. The fusion event occurs in a few steps—firstly, acidification of the endosome causes the dissociation of E1–E2 heterodimers, after which E1 is inserted into the vesicle membrane [53]. As mentioned earlier, E1 plays an important role in mediating membrane fusion—a pH-independent conformational change in the stem region of E1 causes the target membrane to be distorted, allowing for fusion of the two outer leaflets of the membranes to occur. Then, a stable post-fusion E1 homotrimer is formed and refolds, creating a fusion pore through which the viral nucleocapsid is released into the cytoplasm [14,49,51,54].

## 5. Clinical Presentation and Pathogenesis

**Figure 3:**
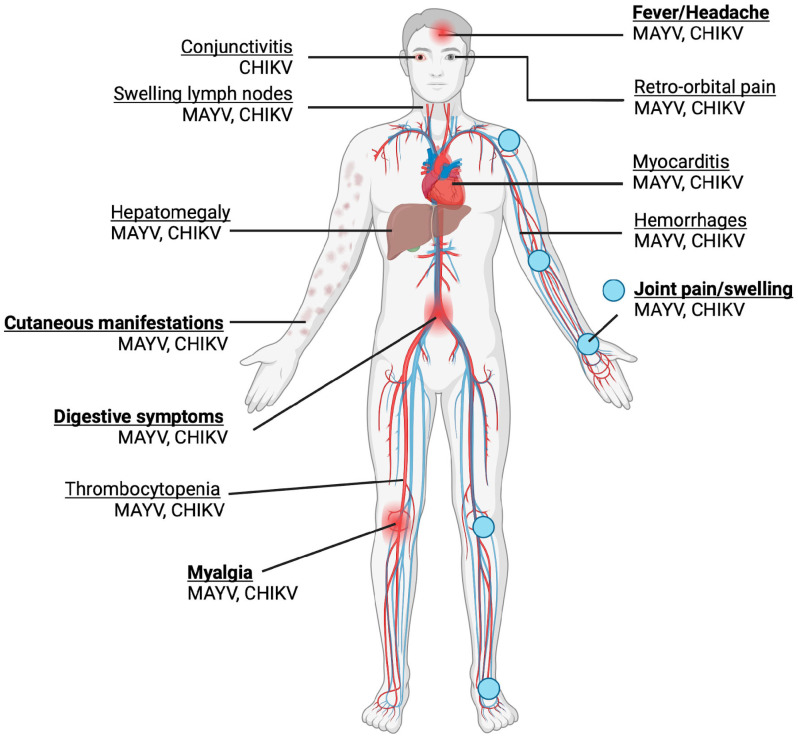
Symptoms of MAYV and CHIKV infection are shown. Acute symptoms are in bold. Joint pain and swelling are the most common symptoms. The majority of the symptoms are shared, with the exception of conjunctivitis, which is associated with CHIKV but not MAYV. (Created with BioRender.com).

MAYV infection generally presents as a mild, self-limited and non-specific febrile illness, sharing significant overlap with the presentation of CHIKV and DENV [9]. Common symptoms include an abrupt fever, which can last 10 days, myalgia, arthralgia, maculopapular rash, fever, headache and diarrhoea, as shown in Figure 3 [55]. Although most MAYV cases are mild, severe complications such as myocarditis and haemorrhagic and neurological manifestations may also occur [9]. Similar to other arthritogenic alphaviruses such as CHIKV, the possibility of developing chronic, severe polyarthralgia makes MAYV infection potentially debilitating, despite no fatalities having been recorded at present, raising significant clinical concern. Among infected patients, an estimated 50–89% and 75% experience arthralgia and myalgia, respectively [56]. MAYV-induced arthralgia and myalgia could persist for a few months to years, with up to 54% of patients experiencing chronic arthralgia at a 12-month follow-up after acute infection [57]. Mainly, the joints affected are those of the hand (63%), knee (38%), wrist (25%), ankle and foot (25%), elbow (13%) and shoulder (7%) [56].

The severity of a Mayaro fever has been found to be linked with the production of pro-inflammatory cytokines and mediators such as MCP-1, IL-2, IL-9, VEGF and IL-17. The association between pro-inflammatory cytokines and mediators has been demonstrated in both human and mice models [10,58,59]. These studies have shown that during the acute phase of Mayaro fever, IL-10, IL-12p70, regulated on activation of normal T-cell expressed and secreted (RANTES) and vascular endothelial growth factor (VEGF) levels are significantly elevated in comparison with healthy controls. During the post-acute arthralgic recovery phase, IL-5-10, IL-13, IL-17, interferon-gamma-induced protein 10 (IP-10), RANTES, macrophage inflammatory proteins 1α and 1β, granulocyte–macrophage colony-stimulating factor and interferon gamma remain significantly elevated [59], suggesting that these mediators contribute to mechanisms leading to persistent arthralgia. This is consistent with the theory that chronic arthralgia is associated with a sustained high expression of pro-inflammatory cytokines. Notably, Santiago et al. (2015) compared the cytokine and chemokine profiles of patients who had fully recovered from acute MAYV infection, patients with chronic arthralgia, and healthy controls [10]. They identified IL-13 as a promising potential mediator of persistent arthralgia due to its sustained high expression in patients with chronic arthralgia but not in fully recovered patients at all studied time points (acute stage, convalescent stage and 3, 6 and 12 months post-infection). Furthermore, IL-7 and VEGF were also identified as potential mediators as they were significantly elevated at all time points in patients with chronic arthralgia but only elevated in the convalescent stage (IL-7), or at 3 and 12 months post-infection (VEGF) in the fully recovered patients [10].

Interestingly, Santiago et al. found that a robust neutralising antibody response was elicited in all MAYV-infected subjects, with no significant difference between the patients with chronic arthralgia and the fully recovered patients [10]. This suggests that a neutralising antibody response alone might not be sufficient to prevent long-term disease outcomes, which has important ramifications for vaccine development, as many vaccine studies use the induction of neutralising antibodies as a primary immune correlate of protection. Overall, studies on immune profiles following MAYV infection to date are relatively few in number and have small sample sizes; hence, further research could better characterise MAYV pathogenesis and identify predictors of severe disease and long-term outcomes, which would help guide the development of vaccines and therapeutics.

## 6. Diagnosis

MAYV testing has not been common practice due to the fact that endemic regions are typically difficult to access and have limited diagnostic laboratory facilities. Diagnosis is further hampered by high rates of false-positive tests due to symptoms of MAYV fever that are shared with related arboviruses [60]. This review will discuss the serological and molecular methods used to detect MAYV infection and their advantages and disadvantages.

### 6.1. Serological Testing

MAYV infection can be diagnosed using serological methods, which detect the presence of anti-MAYV antibodies in patient samples. Methods used to date include haemagglutinin inhibition (HI), complement fixation (CF), plaque reduction neutralisation tests (PRNT), IgM enzyme-linked immunoabsorbent assay (MAC-ELISA) and enzyme immunoassays (EIAs). Commercial kits, in-house assays and lateral flow kits are also available [9,60,61,62,63]. These serological methods are beneficial for seroprevalence testing and for the evaluation of vaccine candidates, but an important limitation of using them for diagnosis is the extent of cross-reactivity between MAYV and related arboviruses, especially those also in the Semliki Forest serocomplex as they are antigenically similar [33,64]. To address this concern, Fischer et al. (2020) studied the specificity of commercially available ELISAs and PRNTs for MAYV-specific and CHIKV-specific sera samples [61]. They found a false-positive rate of around 50% with single ELISA testing, with the cross-reactivity of MAYV-specific sera being 64.7% for IgM and 38.1% for IgG. These results highlight the unreliability of single ELISA testing to diagnose MAYV, but this could be mitigated through parallel ELISA testing for both CHIKV and MAYV, which significantly increased the positive predictive values for all cohorts in the study. PRNTs showed higher specificity (20% cross-reactivity), and the use of PRNT along with ELISA increased the positive predictive values to 100%; however, the routine use of PRNT in the clinic is limited by its cost and labour-intensive process. The high extent of cross-reactivity is particularly significant as these arboviruses co-circulate in overlapping endemic areas; hence, measures such as parallel ELISAs should be established to increase the specificity of serological detection [61], which would improve patient diagnostics and contribute to the accuracy of epidemiological research. Ongoing research to improve serological testing capable of distinguishing MAYV from related arboviruses include the development of alphavirus structural protein microarrays [64] and sensitive MAYV-specific ELISAs [62]. Inspiration could also be drawn from research on other alphaviruses to design similar epitope-blocking ELISAs for MAYV detection [65,66].

### 6.2. Molecular Methods

Molecular techniques targeting viral nucleic acids are also valuable MAYV diagnostic tools. Reverse transcription real-time polymerase chain reaction (RT-qPCR) is considered the gold standard for the molecular detection of MAYV presence due to its high sensitivity and specificity [9,60]. PCR-based molecular diagnostic techniques allow for MAYV detection in patient samples within 3–5 days after infection [60,67]. To date, several RT-qPCR protocols have been described, targeting various genomic sequences such as the 5′ UTR, nsp1 and the E1 gene [7,68,69,70,71]. Specific primers anneal to these sequences, allowing for amplification of the flanked target sequence, then a fluorescent probe is added to enable the real-time detection of PCR products. RT-qPCR results can be further confirmed by nested PCR [69,72]. Multiplex assays can also be used to detect MAYV along with other viruses, enabling differential diagnosis, which is massively beneficial for endemic areas where co-transmission of other arboviruses is common [67]. Naveca et al. (2017) described a multiplexed one-step RT-qPCR method that simultaneously detects MAYV, Oropouche and Oropouche-like viruses, showing that it has high sensitivity (>98% for both targets) and amplification efficiency (limit of detection (LoD): 2–20 copies per reaction) [67]. Another assay for differential diagnosis was described by Wang et al. (2006); this RT-PCR-ELISA assay leverages on the sensitivity of PCR, simplicity of ELISA, and specificity of DNA probes, enabling the differentiation of nsp1 from MAYV and Venezuelan, Eastern and Western equine encephalitis viruses [73]. Importantly, most of the molecular diagnostic tests described to date have not been sufficiently assessed for basic diagnostic parameters, including LoD, analytical specificity, reproducibility and accuracy, using well-defined clinical samples [60]. Therefore, these are important factors to be assessed to validate both existing and novel diagnostic methods in the future.

## 7. Animal Models

Mice models have been critical in aiding the understanding of MAYV pathogenesis as well as evaluating the efficacy of therapeutics [74,75]. Balb/c mice models have improved our understanding of MAYV pathogenesis [58,76]. One study found that Balb/c mice injected with MAYV in the hind-limb footpad exhibited clinical signs of MAYV infection; these clinical signs included ruffled fur, change in gait and eye irritation [58]. Histopathological consequences, such as arthritis and tenosynovitis, were also reported [58]. Non-human primate models such as rhesus macaques have also proved useful in understanding MAYV pathogenesis and immunity [77]. Mice models have helped to identify factors affecting MAYV infection [76]. These factors include the age of mice, the innate immune response and the adaptive immune response. Studies found that young mice below 11 days old inoculated with MAYV in the hind-limb footpad experienced severe weight loss and higher lethality but were better at restricting MAYV infection compared with adult 21-day-old mice [76]. In contrast, adult mice exhibited a higher resistance to MAYV-induced lethality and had a comparable body weight change to controls [76]. Mice models also facilitated the identification of the type I interferon response as a factor affecting MAYV-induced lethality [76]. Figueiredo et al. reported that adult mice lacking type I interferon receptor function (IFNAR^−/−^) had increased expression of pro-inflammatory mediators [76]. H&E staining also found that adult IFNAR^−/−^ mice had sites of injury with necrosis, oedema and infiltration of inflammatory cells at 4 days post-infection. Furthermore, mice models have been crucial in vaccine testing [74,75]. Through the use of Balb/c mouse models, the ability of the live-attenuated MAYV/IRES vaccine to induce a strong cellular and humoral response was identified in mice [74]. Similarly, IFNaR1^−/−^ mice were used to investigate the safety and efficacy of the ChAdOx1 May vaccine [75]. Less commonly used than mouse models, NHPs have also been used in MAYV research. For example, Binn et al. demonstrated that the administration of MAYV antibodies was able to provide cross-reactive protection against CHIKV challenge in an NHP model [78].

## 8. Current Treatments

There are no specific antiviral drugs for MAYV infection, and usually, only symptomatic treatment is recommended [14,57]. Analgesics are administered to relieve pain and fever, including paracetamol and/or non-steroidal anti-inflammatory drugs (NSAIDs), mainly ibuprofen, diclofenac or naproxen [79]. Analgesics are sometimes prescribed for months following acute febrile infection for pain relief of the following debilitating arthralgia. Despite the absence of specific antivirals, there are several drugs being investigated for their potential use in treating Mayaro fever—ribavirin, a drug used to treat CHIKV infection has shown potential for use in Mayaro fever treatment as well due to the similarities in their clinical and pathological features. The exact mechanism of action of ribavirin is undefined, but it has been shown to be a synthetic nucleoside analogue as well as an RNA-dependent RNA polymerase inhibitor [80]. Hence, it possesses broad-spectrum antiviral activity against a range of RNA and DNA viruses. An in vitro study by Biolant et al. (2004) has shown that ribavirin acts synergistically with interferon-alpha, having a cytopathic effect on the MAYV-related alphaviruses CHIKV and Semliki Forest virus (SFV) [81]. Furthermore, the study determined that the compounds 6-azauridine and glycyrrhizin also had antiviral activity against CHIKV and SFV, warranting further research into these compounds as promising future therapeutics against alphaviruses.

Another treatment that has been explored is the anti-malaria treatment chloroquine. In monkey kidney cells, chloroquine treatment has shown in vitro efficacy in reducing MAYV viral yield and restoring cellular protein synthesis while inhibiting viral protein synthesis in a dose-dependent manner [82]. Furthermore, there has also been a case report in which chloroquine therapy successfully treated the chronic arthralgia caused by Chikungunya fever in one patient following unsuccessful cyclooxygenase inhibitor treatment [83]. Given this information, chloroquine may be useful for the treatment of arthralgia following MAYV infection; however, more research should be conducted to establish its clinical efficacy.

As previously discussed, Mxra8 has been identified as a host receptor involved in the entry of MAYV and other arthritogenic alphaviruses, and it therefore presents an attractive therapeutic target. In vivo experiments involving the administration of anti-MXRA8 monoclonal antibodies or the F_c_-Mxra8 fusion protein to mice infected with CHIKV or O’nyong’nyong virus (ONNV) found that both treatments were effective in decreasing viral titres in the ankle and calf muscles as well as reducing footpad swelling, both of which are measures for the evaluation of arthritis and arthralgia in murine models [52]. This highlights the potential role for antibody treatments targeting Mxra8 in the pathogenesis of arthritogenic alphavirus infections [52]. Furthermore, it has been shown that the human mAbs from two donors previously infected with RRV and CHIKV respectively were able to block Mxra8 binding and neutralise several alphaviruses, including MAYV, in vitro, with one of the human mAbs studied also conferring in vivo protection in an immunocompetent mouse model of MAYV infection, highlighting the potential of targeting the Mxra8-alphavirus interaction in developing therapeutics [84]. Interestingly, a study on mouse models found that Fc effector functions were necessary for an effective neutralising antibody response against MAYV infection, reflecting that this is an important consideration for designing antibody-based therapeutics targeting Mayaro fever [85].

## 9. Vaccine Development Efforts

Unfortunately, there are no licensed vaccines as yet, most likely because MAYV remains a neglected tropical disease that circulates within a limited area. Therefore, there have also been few attempts to develop a vaccine. However, several potential vaccine candidates have been investigated: an inactivated vaccine in 1967 [86], a live attenuated vaccine in 2014 [87], a DNA-based vaccine in 2019 [25], as well as two viral-vectored vaccines in 2020 and 2022 [88,89] and two virus-like particle vaccines in 2023 [90].

### 9.1. Vaccine Candidates in Development

The MAYV vaccine candidates in development have been summarised in Table 2 below.

The first vaccine candidate, an inactivated vaccine, used the wild-type MAYV strain TRVL15537, which had been inactivated using formalin [90]. A single vaccination of immunocompetent CD-1 mice was shown to be immunogenic. Furthermore, passive transfer of sera from immunised mice to naive infant mice provided some protection against a lethal MAYV challenge, highlighting that a neutralising antibody response had been elicited by the vaccine [86].

The second vaccine candidate was a live-attenuated vaccine based on a recombinant virus designed by replacing the MAYV internal ribosome entry site (IRES) with the encephalomyocarditis virus (EMCV) IRES [87]. Without the MAYV subgenomic promoter, the expression of structural proteins is reduced, and the resulting phenotype is also unable to replicate in mosquito cells due to inefficient recognition of the EMCV IRES by insect ribosomes, an important feature for a live MAYV vaccine since mosquito transmission of this strain would not be possible. When tested in immunocompetent CD1 and interferon receptor-deficient A129 mice, this vaccine candidate was found to be well-attenuated and also highly immunogenic after a single dose, eliciting an effective neutralising antibody response. It also conferred protection against lethal challenge [87].

A DNA-based vaccine candidate, scMAYV-E, encoding a consensus full-length MAYV envelope gene sequence (including the structural proteins E1, E2 and E3 and the 6K/TF polypeptides) has also been tested [25]. scMAYV-E was tested both in vitro and in vivo; immunisation of interferon alpha/beta receptor knockout (IFNAR^−/−^) mice with scMAYV-E elicited robust anti-E1 and anti-E2 antibody responses, which was also assessed through a plaque reduction neutralisation test (PRNT) as being capable of neutralising MAYV infection of infected Vero cells in vitro. IFN-gamma ELISpot assays also demonstrated that an effective antigen-specific cellular immune response had been elicited. An in vivo murine challenge model also showed that vaccination was able to confer protection against MAYV challenge; furthermore, all naive mice that had received a passive transfer of immune sera survived the challenge, while the naive mice that had received an adoptive transfer of T cells from immunised mice only had partial protection from the disease [25]. Hence, this establishes that the vaccine-induced humoral response was the key component of the protection conferred through vaccination against a MAYV challenge, correlating with previous studies on related alphaviruses, including CHIKV, which highlighted that neutralising antibodies are the primary driver of protection against viral infection [92,93].

The two viral-vectored vaccines which have been explored utilise a recombinant replication-deficient chimpanzee adenovirus vector, ChAdOx [88,89]. ChAdOx1 May expresses MAYV structural proteins. ChAdOx1 May was assessed to have robust immunogenicity, eliciting a high titre of neutralising antibodies, and it conferred in vivo protection in IFNAR^−/−^ mice [88]. ChAdOx2, which is the successor to ChAdOx1 and which has better tolerability and less reactogenicity, has also been investigated as a platform for a MAYV vaccine. ChAdOx2 May showed similar immunogenicity to ChAdOx1 May. ChAdOx2 Chik was also tested in a prime–boost regimen using a single vaccination of ChAdOx2 May followed by a Modified Ankara Virus (MVA) boost encoding the same MAYV structural antigens in BALB/c mice. This elicited a robust neutralising antibody response measured through a virus replicon particle-based neutralisation assay, and the production of anti-E2 antibodies was further confirmed through an ELISA [89].

Recently, MAYV viral-like particle (VLP) vaccines have been investigated. Two VLP- based vaccines were produced from insect or mammalian cells, respectively. Although structurally similar to MAYV, the VLP vaccines do not contain the viral genome. Subsequently, these vaccines have no risk of replicating. In a recent study, Abbo et al. 2023 administered 2 intramuscular injections of 1 μg of the VLP vaccines to mice. Mice received a boost 4 weeks after the first vaccination [90]. At week 10, mice were challenged with Mayaro virus. They found that VLPs derived from either insect or mammalian cells provided mice with complete protection against MAYV genotype L and D challenges. Vaccinated mice were also protected from clinical symptoms of the MAYV disease, including joint inflammation and tendonitis. Moreover, as shown by Sanofi for influenza [94], Novavax for COVID [95] and GlaxoSmithKline for human papillomavirus [91], this vaccine platform has proven efficacy and tolerability. Further investigations regarding the safety and efficacy of these MAYV VLP vaccines in humans are needed.

### 9.2. Is There Potential to Leverage Cross-Protection from CHIKV Vaccination?

As Chikungunya virus (CHIKV) and Mayaro virus have a close phylogenetic relationship, the possibility of cross-protection being elicited by CHIKV vaccines has been proposed. Webb et al. (2019) explored the effects of CHIKV immunity on susceptibility to MAYV in mice [96]. In mice pre-exposed to wild type CHIKV, strong cross-protection against MAYV was reported. Subsequently, MAYV spread could be restricted due to the pre-existing CHIKV immunity in some regions. The study showed that mice vaccinated with CHIKV/IRES and MAYV/IRES demonstrated comparable levels of protection from disease, viremia and death upon a MAYV challenge [96]. In contrast, mice vaccinated with EILV/CHIKV, another potential CHIKV vaccine candidate, displayed a lack of protection against MAYV disease or death [96].

Campos et al. (2020) also conducted experiments to investigate the extent of cross-reactive protection in CHIKV- and MAYV-vaccinated mice [88]. They found that only partial cross-protection was conferred against heterologous viral challenges in A129 mice. Following MAYV challenge, mice vaccinated with ChAdOx1 May or MAYV-IRES survived and did not experience significant weight loss or foot swelling, whereas mice vaccinated with ChAdOx1 Chik showed significantly decreased viraemia but were only partially protected against weight loss (two out of five mice), and all five mice were not protected against foot swelling [88]. These studies highlight interesting insights about the potential for CHIKV vaccines to provide cross-protection against MAYV. In light of the recent licensure of Ixchiq, a live-attenuated CHIKV vaccine [97], studies investigating the potential cross-protection elicited by Ixchiq should be explored. As well as cross-reactive protection against MAYV from CHIKV vaccines, a recent paper has explored the potential provided by a trivalent vaccine containing a mixture of encephalitic virus-like particles (VLPs) [98]. Sutton et al. immunised mice with a trivalent vaccine containing Western, Eastern and Venezuelan equine encephalitis VLPs. They found that this vaccine was able to induce triple-specificity monoclonal antibodies, namely SKT05 [98]. Further studies in vivo demonstrated that SKT05 provided protection against both arthritogenic alphaviruses, like MAYV, and encephalitic alphaviruses. This study highlights the potential of cross-reactive protection against multiple alphaviruses conferred by vaccine-elicited antibodies.

## 10. Conclusions

In this review, we described an emerging arbovirus, MAYV, with a particular focus on treatments and vaccine development efforts. To conclude, MAYV poses a major threat to global health, which should not be overlooked simply due to its area of distribution. Importantly, there exists a great risk that the prevalence of MAYV may increase in the future due to climate change and the widening distribution of its competent vectors. To ensure that any future spread can be appropriately tracked and prevented from developing into an epidemic, it is vital that more accurate diagnostic measures and specific treatments be developed. Moreover, further research should be undertaken in order to develop effective vaccines against MAYV. This review has highlighted promising vaccine candidates which have been investigated, and the roll-out of a successful vaccine in the future could play an important role in limiting the spread of MAYV.

## Figures and Tables

**Table 1:** Functions of the non-structural proteins (nsPs).

Non-Structural Protein	Function
Non-structural protein 1 (nsP1)	Involved in mRNA capping as it has guanine-7-methyltransferase and guanylyl transferase activities. Potential role in anchoring viral replication complexes.
Non-structural protein 2 (nsP2)	Involved in DNA replication as N-terminus has helicase, RNA-dependent 5’-triphosphatase and nucleoside triphosphatase activity. Involved in cleaving the non-structural polyprotein as it has cysteine protease activity.
Non-structural protein 3 (nsP3)	Involved in DNA replication as it contains 3 domains needed for replication.
Non-structural protein 4 (nsP4)	Involved in viral replication as it contains RNA-dependent RNA polymerase activity.

**Table 2:** Summary of published studies on MAYV vaccine candidates.

Vaccine	Technology	MAYV Immunogen	Development Stage	Efficacy	Advantages	Disadvantages	Reference
ChAdOx1 May	Adenovirus-vectored vaccine	C, E3, E2, 6K and E1	Preclinical	In an A219 mice model, ChAdOx1 May provided complete protection against a MAYV challenge and induced high titres of neutralising antibodies.	Non-replicating	Efficacy could be affected by prevalence of pre-existing immunity in community	Campos et al. (2020) [88]
ChAdOx2 May	Adenovirus-vectored vaccine	C, E3, E2, 6K and E1	Preclinical	BALB/c mice vaccinated with 1 × 10^8^ infectious units per animal of ChAdOx2 May followed by an MVA boost elicited high titres of anti-MAYV E2 antibodies.	Higher tolerability and less reactogenicity compared to ChAdOx1 May Non-replicating	Efficacy can be affected by prevalence of pre-existing immunity in community	Kim et al. (2022) [89]
MAYV/IRES	Live-attenuated	Recombinant virus: subgenomic promoter is replaced by IRES	Preclinical	Mice vaccinated with MAYV/IRES had fewer clinical outcomes associated with MAYV disease. Specific anti-MAYV IgM and IgG responses were found to be greater in vaccinated animals.	Well-established technology	Replicating Cold chain storage required	Mota et al. (2020) [74] Webb et al. (2019) [91] 14/08/2024 15:15:00
scMAYV-E	DNA vaccine	Synthetically designed consensus MAYV envelope sequence	Preclinical	In mice, scMAYV-E was found to induce strong humoral and T-cell responses. Following MAYV challenge, vaccinated mice were fully protected from clinical signs of disease and mortality.	Stable at warm temperatures, could reduce the need for cold chain storage Non-replicating	Risk of genomic integration	Choi et al. (2019) [25]
MAYV-VLP	Virus-like particles	Structurally similar to MAYV but does not contain viral genome	Preclinical	A challenge study in mice found that a single dose of MAYV-VLP was sufficient to elicit high neutralising antibody titres. Two doses of MAYV-VLP were found to provide complete protection.	Adjuvant is often not needed Non-replicating	High production costs	Abbo et al. (2023) [90]

## Data Availability

Not applicable.
